# Population structure and genetic diversity in *Eucalyptus pellita* based on SNP markers

**DOI:** 10.3389/fpls.2023.1278427

**Published:** 2023-12-15

**Authors:** Chubiao Wang, Jun Lan, Jianzhong Wang, Wenliang He, Wanhong Lu, Yan Lin, Jianzhong Luo

**Affiliations:** ^1^ Research Institute of Fast-growing Trees, Chinese Academy of Forestry, Zhanjiang, China; ^2^ Forestry Science Research Institute, Guangxi Dongmen Forest Farm, Fusui, China

**Keywords:** *Eucalyptus pellita*, single nucleotide polymorphism, population structure, genetic diversity, population differentiation

## Abstract

*Eucalyptus pellita* has the characteristics of rapid growth and high resistance. However, there is little research on molecular breeding of *E. pellita*, which is essential to shortening breeding life and selecting quality varieties. Therefore, a crucial step before selective breeding can be carried out to increase the wood quality of *E. pellita* is identifying genetic diversity and population structure using single nucleotide polymorphism (SNP) markers. In this study, the genetic diversity of 1^st^ generation 196 *E. pellita* families from 23 geographically defined was assessed using 1,677,732 SNP markers identified by whole genome resequencing. SNP annotation showed that the ratio of non-synonymous to synonymous coding mutations was 0.83. Principal component analysis (PCA), phylogenetic tree, and population structure analysis permitted the families to be categorized into three groups, one of which (G2) contains most of the Indonesian (IDN) and Papua New Guinea (PNG) families. Genetic relationship analysis showed that IDN was closely related to PNG. Genetic diversity analysis showed that He, PIC, I, and H mean values were 0.2502, 0.2027, 0.3815, and 0.2680, respectively. PCA analysis classified various provenances in QLD into two categories (G1 and G3). The genetic diversity of G3 was higher than that of G2. The results of genetic differentiation (Fst) showed that PNG region was divided into two groups (PNG1 and PNG2), the Fst (0.172) between QLD and PNG2 region was higher than QLD and PNG1, and the Fst (0.024) between IDN and PNG1 is smaller than IDN and PNG2. A Mantel test revealed a positive correlation between the genetic and geographic distance of *E. pellita*. This study has a certain reference value for genetic identification, germplasm preservation, and breeding of *E. pellita*. Also, it provides a basis for subsequent association analysis to explore excellent alleles and introduction.

## 1 Introduction

*Eucalyptus pellita*, a fast-growing tree with strong disease resistance and a high survival rate, has been introduced and cultivated in numerous countries, such as China, Brazil, and Western Samoa. *E. pellita* thrives after introduction since the cultivated climate is comparable to that of the native distribution area. Currently, Australia (Brawner et al., 2010), Indonesia (Leksono et al., 2008), and Vietnam (Harwood and Nambiar, 2014) have advanced *E. pellita* seed orchards to the second or even the third generation. The average annual yield of *E. pellita* is 16-18 m^3^/ha in Sumatra, Indonesia, while the average annual yield of *Eucalyptus* in China is 15-28 m^3^/ha (Harwood and Nambiar, 2014). Before 2014, *Eucalyptus* plantations in China were in a developmental and exploratory stage. Many individuals and investors recognized the economic benefits of *Eucalyptus* and ventured into *Eucalyptus* forestry. However, the problem of poor *Eucalyptus* varieties also limited *Eucalyptus* yields. According to data from 2009 to 2018, domestic *Eucalyptus* annual yields have increased rapidly, reaching as high as 39.43 m^3^/ha. The main reason for this increase is that China began to place significant emphasis on the development of artificial *Eucalyptus* forests.

*E. pellita* is a humid and subtropical forest species. It has two naturally occurring regions: southern New Guinea (NG) and North Queensland (QLD). *E. pellita* was first discovered in 1864 by John Dallachy at Rockingham Bay, south of Innisfail in Queensland, and described in the same year by Baron Ferdinand von Mueller (Harwood, 1998). By the end of the 1980s, *E. pellita* was thought to be limited to Australia, with widespread distribution in northern QLD and New South Wales. Similar to *E. pellita*, *Eucalyptus Scias* is found in Lanzhou, New South Wales, however, its descriptions of leaves, buds, and fruits are very different from that of the species (Johnson and Hill, 1990). The *E. pellita* of Cape York in northern QLD are similar to the NG population and in some respects have fewer fruits and leaves than the southern Australian population. Further research may admit that the *E. pellita* populations in NG and Cape York in QLD originated independently of each other (Harwood, 1998). However, few studies on genetic diversity and population structure of *E. pellita* have been reported, which is of great significance in distinguishing the relationship between the three provenances.

Initially, a large number of introduced species and high-generation improvements were carried out in Brazil, Southeast Asia, and other countries, with the primary goal of studying the genetic variation of growth, adaptability, wood characters, disease resistance, and genetic gain between generations (Brawner et al., 2010). The majority of the genetic materials investigated were from natural sites, and phenotypic traits analysis effectively distinguished between QLD and NG provenances. In plantings of *E. pellita* in eastern Colombia, the provenance of NG performed noticeably better than that of QLD (Nieto et al., 2016). Similarly, when planted in humid tropical environments, the NG outperformed the QLD in terms of survival rate, growth rate, and morphological characteristics (Harwood et al., 1997). In addition, the internal provenance of QLD and NG was also different. For the survival study of introduction, *E. pellita* was introduced to Urbano Santos in Brazil. The survival rates of Northeastern Coen and South Helenvale at 3.5 years of age were 29.8% and 43.8%, respectively (Harwood, 1998). Although the geographical location of the various provenances is established, the necessity for genetic relationships between provenances remains to be determined.

In China, *E. pellita* is mostly utilized as a hybrid parent to develop excellent *Eucalyptus* clones and to conduct theoretical research on cross-breeding for fast growth, disease resistance, and insect pests. Long-term artificial selection and domestication also introduce a slew of issues, the most prominent of which is the limited genetic diversity of breeding materials, which severely homogenizes variations and makes it harder and harder to produce novel, ground-breaking varieties (Zhang et al., 2012). The selection of parents in cross-breeding is aided by knowing the genetic background of the breeding materials; this increases the effectiveness of producing new varieties with evident heterosis.

Genetic diversity plays an important role in heterosis and breeding programs. Therefore, the genetic diversity within and between *Eucalyptus* populations is routinely assessed using different marker techniques such as morphological (Byrne et al., 2016), biochemical (Kirst et al., 2005), and molecular markers (Shang et al., 2019). Morphological markers have been extensively utilized to assess genetic diversity because they are inexpensive, fast, and easy to measure. They are, however, highly influenced by the environment, and several other factors limit their ability to estimate genetic diversity (Yaman, 2021). Because molecular markers are stable, polymorphic, easily obtainable in the genome, and insensitive to environmental factors, they are still valuable tools for measuring genetic diversity (Govindaraj et al., 2015). Therefore, molecular markers, including RFLP, RAPD, ISSR, SSR, and SNP based on single nucleotide differences, are the most ideal methods to explain biological genetic diversity.

The study of genetic diversity and population structure is essential in identifying genetic relationships among germplasm resources. Plant improvement initiatives benefit from genetic heterogeneity among populations as well as genetic relationships between them. Identification of populations with high levels of genetic variation will be a valuable resource for broadening the genetic base because it makes it possible to identify good alleles for traits (Yang et al., 2020; Aesomnuk et al., 2021). To comprehend and use accessible gene bank resources, a variety of approaches can be employed to identify *E. pellita* genetic diversity. For example, early isoenzyme markers technology discovered considerable genetic variations between NG and QLD provenances, with NG provenances having much lower heterozygosity (House and Bell, 1996). Similarly, RFLP was also used to evaluate the genetic resources of *Eucalyptus*, though the results were stable, reliable, and repeatable (Moran et al., 2000). However, it has some drawbacks, such as complicated operation, long duration, high cost, and large demand for DNA.

So far, studies on *E. pellita* based on SNP markers are scarce, and earlier techniques are prohibitively expensive and may yield inaccurate results. Therefore, SNP markers are being promoted with the advent of next-generation sequencing (NGS) technology. SNP molecular markers have been widely used to study genetic diversity due to their richness, wide genomic coverage, availability of neutral variation and selected loci, rapid and high-yield genotyping, and low error rate (Helyar et al., 2011). Based on SNP markers, complex traits were anatomized by QTLs (Quantitative traits Loci) or LD (linkage disequilibrium) analysis to provide a basis for *Eucalyptus* breeding (Resende et al., 2017; Butler et al., 2022).

Whole-genome resequencing is the process of sequencing the entire genome sequence of different individuals or species with known genome sequence, to analyze the differences between different populations or individuals (Risch and Merikangas, 1996). A large number of SNP, InDel, and SV can be discovered by comparing the sequenced sequence of a single individual with the known genome sequence of that species or related species (Catanach et al., 2019). In this study, 196 *E. pellita* resources from New Guinea and Australia were sequenced using whole-genome resequencing technology, and SNP sites were detected, screened, and typed. Genetic diversity, population structure, and genetic differentiation of these materials were further analyzed. It will lay the foundation for conservation and utilization of *Eucalyptus* resources, gene mapping of important traits, polymeric breeding based on molecular markers and further genome-wide association analysis.

## 2 Materials and methods

### 2.1 Germplasm

The provenances of the 1^st^ generation of *E. pellita* breeding populations are from Queensland (QLD, AUS) and New Guinea (NG) Island, which are separated by the ocean with 150 km as the closest shoreline. Generally, the provenances of NG are divided into Indonesian provenances (IDN) and Papua New Guinea provenances (PNG). QLD is divided into Cape York provenances and northeast Queensland provenances. In this study, 196 *E. pellita* germplasm resources were collected, including 5 from IDN, 55 from PNG, and 133 from QLD. All seeds were randomly planted in Fusui, Guangxi, China, and Suixi, Guangdong, China. Each family was divided into 23 provenances according to geographical location, as detailed in **Supplementary Table** **S1**. After gathering young leaves from the top canopy of surviving trees in mature forests, the leaves were swiftly placed in an incubator with ice for sample and sealed in a zippered bag containing silica gel. The samples were brought indoors and stored in the refrigerator at -24°C.

### 2.2 DNA extraction

The CTAB method was used to extract DNA (Gan et al., 2003). Use Nanodrop2000 Spectrophotometer (Thermo Fisher Scientific) to determine the concentration and quality of the total genome DNA. DNA libraries with a mean insert size of 350 bp were constructed, and 125-bp paired-end reads were generated using an Illumina HiSeq 4000 instrument. Library preparation and sequencing were carried out at the Biomarker Technologies Corporation (Beijing, China).

### 2.3 SNP and InDel calling

Illumina platform was used for sequencing, and Raw image data files were identified by CASAVA bases to form Raw reads. Subsequently, Clean reads were obtained through quality control analysis, base quality distribution analysis, and sequencing data filtering. BWA software (Li and Durbin, 2009) was used to compare Clean reads with *E.grandis* genomes (https://www.ncbi.nlm.nih.gov/assembly/GCF_016545825.1), and the results were formatted by SAMTOOLS software (Li et al., 2009), and then reweighted by PICARD software (http://broadinstitute.github.io/picard/;v1.94). The ratio of pairs, genome coverage distribution, insert-size distribution, and variation were also analyzed. SNPs and InDels within the 196 families were called using the HaplotypeCaller module in GATK (McKenna et al., 2010). They were filtered with the following parameters: QD < 2.0 || MQ < 40.0 || FS > 60.0 || QUAL < 30.0 || MQrankSum < -12.5 || ReadPosRankSum < -8.0 -clusterSize 2 -clusterWindowSize 5. SNPs with minor allele frequency (MAF) of lower than 5% in the population were filtered out. Software SnpEff (Cingolani et al., 2012) was used to annotate variation and predict the impact of variation. The position of mutation locus on the reference genome can be obtained by SnpEff analysis and functional annotation.

### 2.4 Population structure analyses

Based on the genetic data of the population, the genetic distance was calculated and phylogenetic tree was constructed based on the distance matrix. MEGA5.2 (Tamura et al., 2011) software was used to construct phylogenetic trees for each sample, with the Kimura 2-parameter model, 1000 bootstrap replicates, and neighbor-joining method.

EIGENSOFT was used for principal component analysis to obtain sample clustering (Price et al., 2006). Principal component analysis (PCA) analysis can be used to know which sample relationships are relatively close and which sample relationships are relatively distant and can play an auxiliary role in evolutionary analysis.

Admixture software was used to analyze and study the population structure (Alexander et al., 2009). The pre-set subgroup number (K value) of the population was clustered, with a range from 1 to 10. The optimal clustering number was determined according to the minimum value of cross-validation error rate.

GCTA software was used to estimate the genetic relationship between two individuals in natural populations (Yang et al., 2011). In this study, the mean of the expected variances of SNP markers was used to correct the labeled expectation variances, namely the G-matrix, to obtain a heat map of kinship.

### 2.5 Genetic diversity and LD analysis for 23 provenances

Nei’s genetic diversity index (H), polymorphic information content (PIC), minor allele frequency (MAF), expected heterozygosity (He), and observed heterozygosity (Ho) of the 23 provenances and inferred groups were calculated by PowerMarker V3.25 (Liu and Muse, 2005). PopGenome R package (Pfeifer et al., 2014) was used to calculate the nucleotide polymorphism (Pi) and Tajima’ D for 23 provenances. GenAlEx V6.5 (Peakall and Smouse, 2006) was used to calculate the pairwise genetic differentiation coefficient (Fst) between provenances to study population differentiation. PopLDdecay (Zhang et al., 2019) was used to evaluate LD value and plot LD decay for each chromosome.

### 2.6 AMOVA analysis, Mantel test, and genetic difference for three population

Based on Qual > 30,000 in the original data, 12,475 SNP markers were selected for subsequent analysis. R language was used to generate SNP format 0,1,2 from the filtered data, where 0 represented homozygote reference,1 represented heterozygote, and 2 represented homozygote substitution. The GenAIEx V6.5 software (Peakall and Smouse, 2006) was used to perform AMOVA to divide the total level of genotypic variance into variance within and between populations. The Mantel test was used to correlate the genetic distance and geographic distance matrices between populations by GenAIEx V6.5 software. In addition, these filtered SNPs were used to perform Fst and Nei’s genetic distance analysis for three populations by GenAIEx V6.5 software (Fufa et al., 2022).

## 3 Results

### 3.1 Resequencing 196 materials of *E. pellita*

**Figure 1:**
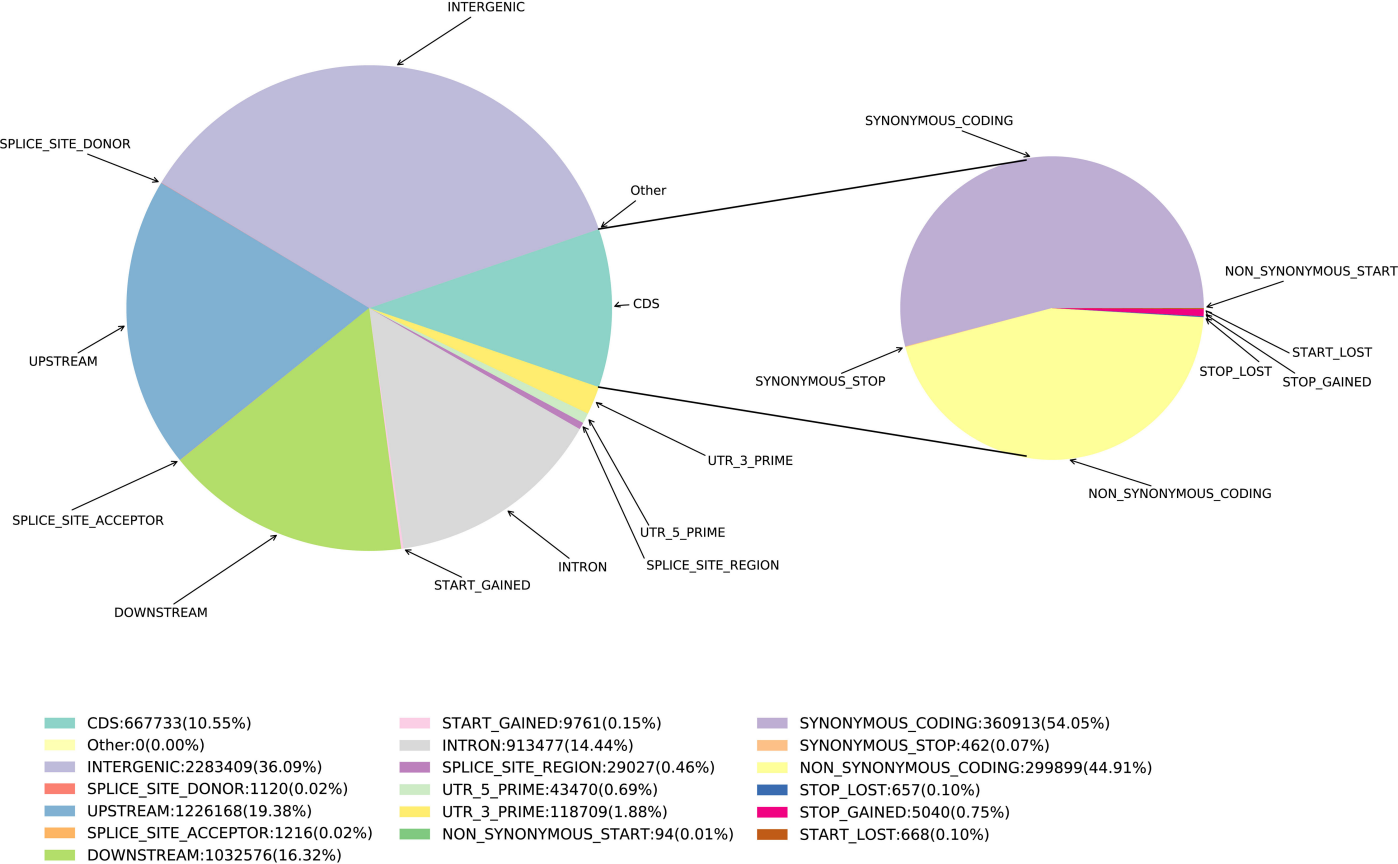
SNP annotated results. The pie chart on the right is based on the CDS region.

Based on SNPs data, we investigated the polymorphism distribution in *E. pellita* genome regions and found that 242,265 (14.44%), 325,144 (19.38%), 273,806 (16.32%) and 605,493 (36.09%) SNPs were located in intron and upstream region (within 5 kb upstream of transcription start site), downstream region (within 5 kb downstream of transcription stop site) and intergenic region (**Figure 1**), respectively. In coding regions (The number of SNPs is 177,001), we annotated 79,491 non-synonymous,177 start-lost, 1,328 stop-gained, and 124 stop-lost SNPs, which led to amino acid changes, longer transcripts, or premature stop codons. In addition, 95,669 SNPs in the coding region were synonymous mutations, and the ratio of non-synonymous to synonymous SNPs was 0.8309.

**Figure 2:**
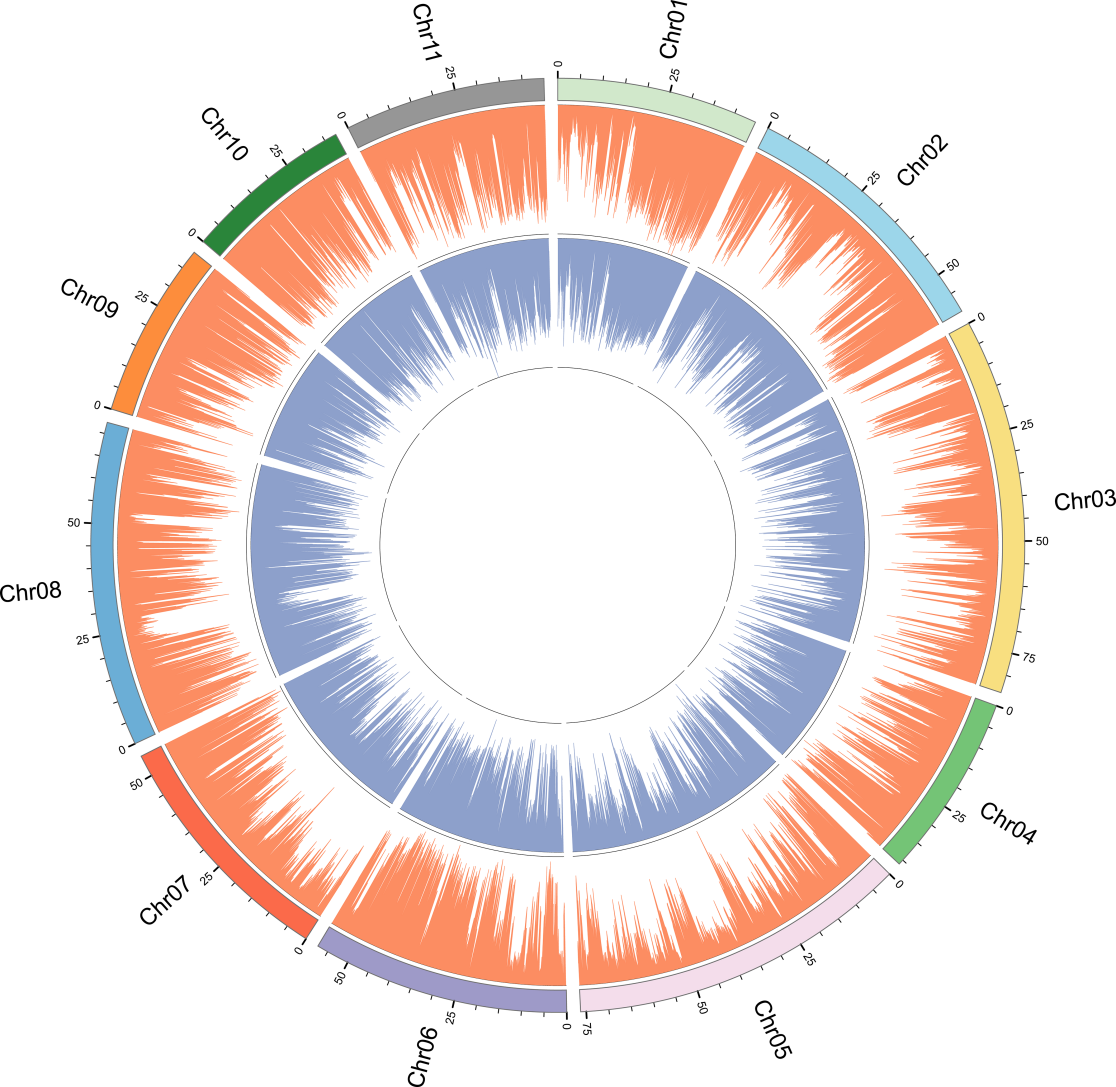
SNP and InDel distribution plot for genotyping-by-sequencing of 196 *E. pellita* accessions. SNP and InDel are represented by orange and blue bars.

In order to determine whether there are regions with high genetic differentiation, we performed chromosomal localization of SNPs and InDels loci (**Figure 2**). It can be seen that the density trend of SNPs and InDels sites is basically the same, showing a radial distribution. For example, the genetic differentiation degree of SNPs and InDels was relatively low around the 25 Mbp interval of chromosome 8. Furthermore, there was a substantial low genetic differentiation region in the middle of each chromosome, with SNP sites being particularly prominent. Moreover, high levels of genetic differentiation were not uniformly distributed across different chromosomes; for instance, chromosomes 1 and 7 exhibited a concentration of differentiation at their ends, whereas other chromosomes displayed uneven variations.

### 3.2 Population structure analysis

**Figure 3:**
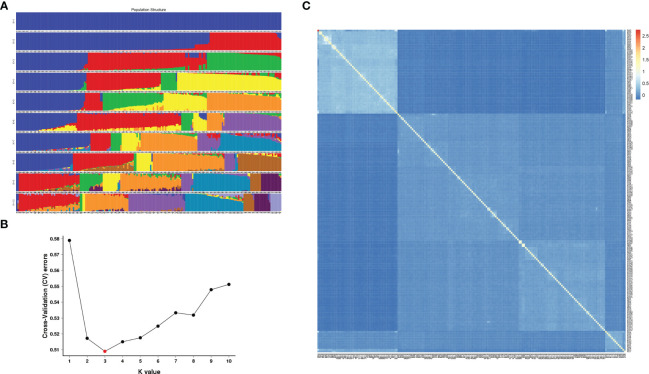
Population structure and genetic relationship. **(A)** In each population structure, each individual was represented by a line of different colors, and which subgroup the variety belonged to was inferred according to the proportion of colors; **(B)** Cross-validation error rate for each K value; **(C)** The smaller the value of kinship between the two samples, the darker the blue shaded part.

According to integrity > 0.8 and MAF > 0.05, all the above SNP markers were filtered, and a total of 1,677,732 SNPs with high consistency were obtained. Based on the SNPs filtered, admixture software was used to analyze the population structure of the samples, and the sample population number (K value) was assumed to be 1-10 for clustering (**Figure 3A**). According to the valley value of cross-validation error rate, the optimal population number was 3, indicating that 196 *E. pellita* families can be divided into three groups, which means they come from different original ancestors. The clustering with K values ranging from 1 to 10 and the cross-validation error rate corresponding to each K value is shown in **Figure 3B**. The population structure divided the families into Q1-Q3 (**Supplementary Table** **S2**), and most of the families from PNG and IDN were assigned to Q1. Families from the QLD region included 85 in Q2 and 47 in Q3.

As can be seen from the kinship heat map of *E. pellita* group (**Figure 3C**), as a natural group, the kinship between the families of the 1^st^ generation of *E. pellita* was not close and their kinship value was mostly low. However, the families in the upper left corner of the figure were relatively close, and most of these families come from the PNG region. In the white-blue shade in the upper left corner, PNG had 45 families, IDN had 4 families, and QLD had 2 families (**Supplementary Table** **S3**), which meant that the genetic distance between these 51 families was close. It also implied that IDN was closely related to PNG region.

**Figure 4:**
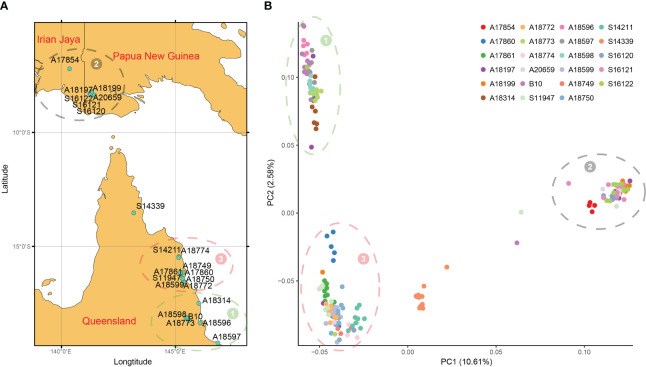
Geographical distribution and PCA. **(A)** Geographic distribution of 23 provenances in this study; **(B)** The first two principal component principal component analyses (PCA) based on genome-wide SNP data. All provenances can be divided into three groups, and the location of each group is consistent with the geographical distribution, represented by numbers 1 to 3 respectively.

To further verify the accuracy of the above results, principal component analysis and cluster analysis were performed on SNP data. According to the geographical location of each family (**Figure 4A**) and the distribution of each family in PCA (**Figure 4B**), they could be divided into 3 groups, namely, group 1-3. 196 families from 23 provenances clustered into 3 groups and only provenances S14339 were relatively discrete and did not belong to the 3 groups, indicating that this population mainly came from 3 branches. For example, the provenances from IDN and PNG belong to Group 2 and are relatively independent from other provenances. The families of Group 2 are concentrated in NG Island (5°S-10°S), and the provenances from QLD are also divided into two groups according to latitude. Group 3 is located between 15°S and 17°S, and Group 2 is located in an area above 17°S (**Figure 4A**). Provenances from different regions have specificity and can be distinguished, and the relationship between provenances and SNP can be better understood by combining the traits of provenances from the three regions.

**Figure 5:**
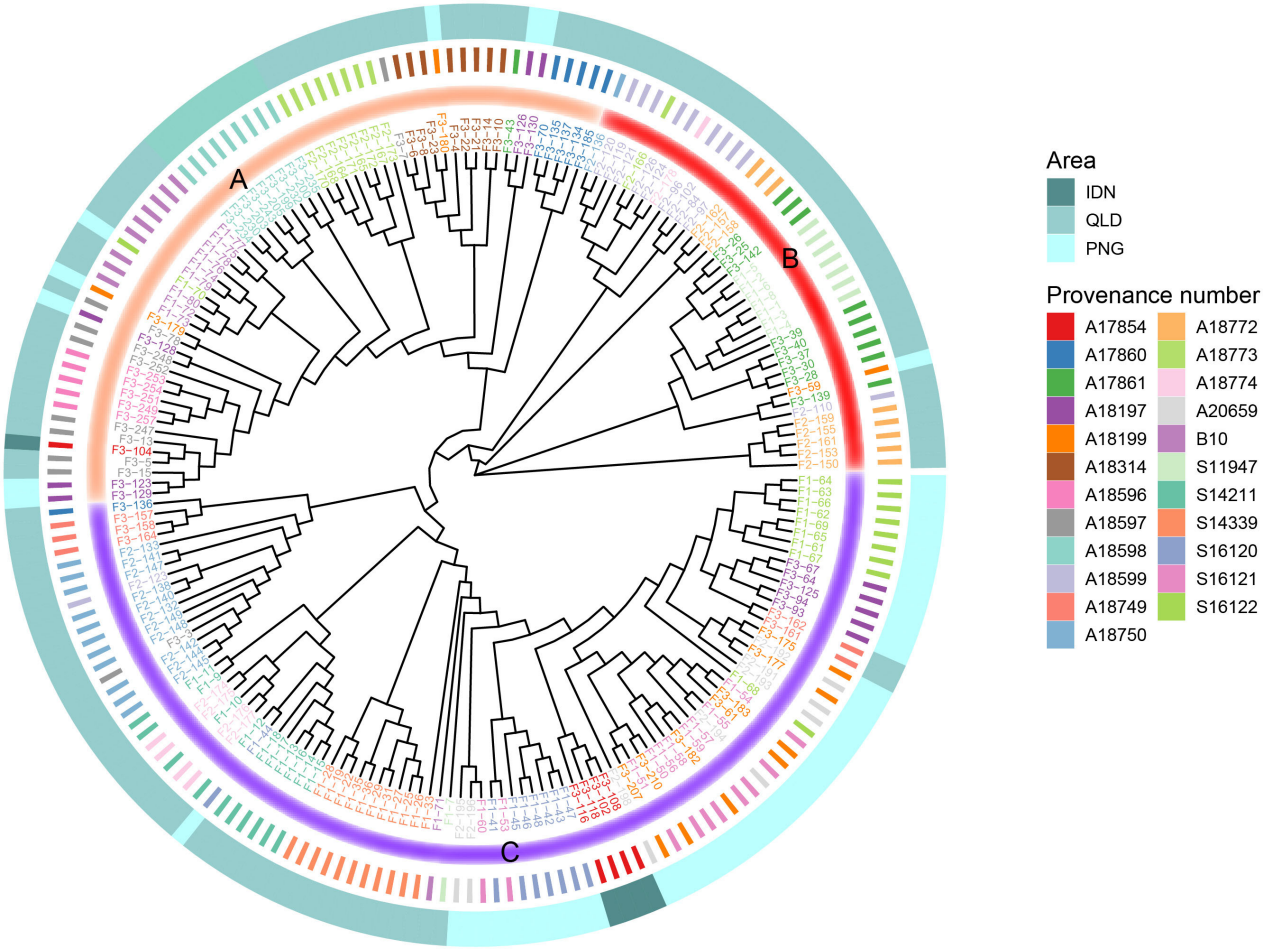
Phylogenetic tree of the 196 *E. pellita* families based on SNPs. The outermost circle indicates the region to which each family belongs, 3 different regions are represented by different colors, and the innermost circle divides each family into three categories Group A, B and C, which are represented by different colors.

To better understand the clustering of each family, we constructed a phylogenetic tree for 196 families (**Figure 5**). From the roots of the developing tree, all provenances can be divided into three large clades (Group A-C). As mentioned above, PNG and IDN were geographically relatively close to each other, both on the island of NG. It can be seen that IDN families were classified into Group C (except F3-104), of which most PNG families were classified into Group C. In addition to PNG and IDN provenances in Group C, some QLD provenances were also classified in Group C, including provenances S14339, S14211, S18774, and A18750. Most of the families of Group A branch were located in southern QLD, while most of the families of Group B originated from northern QLD.

### 3.3 Genetic diversity analysis

According to the genetic diversity parameters of the first-generation *E. pellita* population (**Table 1**), the overall expected heterozygosity of all provenances was 0.2502. The expected heterozygosity of QLD, PNG, and IDN were 0.2547, 0.2428, and 0.2232, respectively. The provenance with the highest expected heterozygosity was A18197 while the smallest provenance was S16120 with little difference among provenances. The observed heterozygosity of provenances A17854, A17860, A18314, A18596, A18598, A18774, and A20659 was higher than the expected heterozygosity, suggesting that these populations may have experienced distant hybridization. The Nei’s diversity index, Shannon-Wiener index, and polymorphism information content of all provenances were 0.2680, 0.3815, and 0.2027, respectively. They all follow the same pattern, with the highest values in the QLD region, followed by the PNG region, and the lowest values in the IDN region. Overall, the genetic diversity is highest in the QLD region and lowest in the IDN region. The highest genetic diversity was A18197 and A18599, and the lowest was A17854 and S16120. In the population predicted by PCA (**Figure 4**), the genetic diversity of Group 3 was greater than that of Group 1, and that of Group 1 was greater than that of Group 2, of which Group 1 and Group 3 were from the QLD area. In addition, the average nucleotide polymorphism across all sources is 0.000669, indicating that the 23 germplasm sources from the three major regions have not undergone artificial selection. This is consistent with their natural population attributes. Tajima’s D test analysis also suggests the presence of a higher frequency of low-frequency variants within various provenances, possibly due to the imbalance caused by genetic drift or selection.

**Table 1:** Genetic diversity parameters of various provenances and inferred groups of *E. pellita*.

Provenances/Group/Area	Na	Ne	MAF	He	Ho	PIC	I	H	Pi	Tajima’s D test
A17854	1.6627	1.3717	0.2477	0.2232	0.2260	0.1806	0.3388	0.2493	0.000622	0.121
A17860	1.7587	1.4291	0.2487	0.2564	0.2683	0.2071	0.3882	0.2808	0.000701	0.409
A17861	1.8305	1.4378	0.2314	0.2636	0.2551	0.2137	0.4027	0.2781	0.000694	0.723
A18197	1.8642	1.4457	0.2264	0.2701	0.2514	0.2196	0.4141	0.2850	0.000711	0.646
A18199	1.8579	1.4299	0.2201	0.2620	0.2320	0.2136	0.4035	0.2765	0.000690	0.536
A18314	1.7813	1.4220	0.2373	0.2533	0.2594	0.2051	0.3858	0.2709	0.000676	0.569
A18596	1.6511	1.3884	0.2635	0.2294	0.2538	0.1843	0.3446	0.2562	0.000639	0.365
A18597	1.8003	1.4243	0.2329	0.2554	0.2539	0.2070	0.3899	0.2711	0.000677	0.630
A18598	1.7924	1.4228	0.2344	0.2540	0.2622	0.2057	0.3873	0.2695	0.000673	0.650
A18599	1.8408	1.4354	0.2271	0.2633	0.2570	0.2138	0.4031	0.2764	0.000690	0.760
A18749	1.7380	1.4291	0.2559	0.2556	0.2452	0.2061	0.3857	0.2855	0.000713	0.269
A18750	1.8324	1.4265	0.2247	0.2578	0.2402	0.2094	0.3952	0.2696	0.000673	0.800
A18772	1.7773	1.4227	0.2389	0.2536	0.2495	0.2052	0.3859	0.2714	0.000678	0.605
A18773	1.8086	1.4344	0.2357	0.2609	0.2604	0.2113	0.3976	0.2771	0.000692	0.687
A18774	1.7036	1.4087	0.2561	0.2433	0.2601	0.1961	0.3671	0.2717	0.000678	0.260
A20659	1.6964	1.3879	0.2450	0.2319	0.2414	0.1874	0.3518	0.2508	0.000626	0.545
B10	1.8141	1.4214	0.2275	0.2541	0.2501	0.2062	0.3891	0.2682	0.000669	0.641
S11947	1.8225	1.4378	0.2338	0.2636	0.2630	0.2137	0.4025	0.2821	0.000704	0.515
S14211	1.8096	1.4221	0.2289	0.2546	0.2537	0.2065	0.3894	0.2686	0.000671	0.677
S14339	1.8167	1.4272	0.2296	0.2570	0.2493	0.2083	0.3927	0.2699	0.000674	0.787
S16120	1.7075	1.3671	0.2284	0.2217	0.2157	0.1801	0.3398	0.2377	0.000594	0.416
S16121	1.7380	1.4000	0.2375	0.2401	0.2195	0.1943	0.3653	0.2537	0.000633	0.854
S16122	1.7563	1.3808	0.2213	0.2308	0.2159	0.1877	0.3549	0.2437	0.000608	0.536
Mean	1.7766	1.4162	0.2362	0.2502	0.2471	0.2027	0.3815	0.2680	0.000667	0.547
IDN	1.6627	1.3717	0.2477	0.2232	0.2260	0.1806	0.3388	0.2493	–	–
PNG	1.7701	1.4019	0.2298	0.2428	0.2293	0.1971	0.3716	0.2579	–	–
QLD	1.7861	1.4244	0.2379	0.2547	0.2551	0.2062	0.3879	0.2729	–	–
Group 1 (G1)	1.7746	1.4189	0.2386	0.2512	0.2566	0.2033	0.3824	0.2688	–	–
Group 2 (G2)	1.7547	1.3976	0.2323	0.2400	0.2288	0.1948	0.3669	0.2567	–	–
Group 3 (G3)	1.7904	1.4277	0.2384	0.2569	0.2547	0.2080	0.3911	0.2760	–	–

Na, observed number of alleles; Ne, expected number of alleles; MAF, minor allele frequency; Ho, observed heterozygosity; He, expected heterozygosity; H, Nei’s diversity index; I, Shannon-Wiehner index; PIC, polymorphism information content; Pi, nucleotide polymorphism; “-”, the value is not calculated.

### 3.4 Population differentiation analysis

The population differentiation analysis of the 1^st^ generation of *E. pellita* population (**Table 2**) showed that the average Fst of IDN and PNG was only 0.039, while the average Fst of IDN and QLD was 0.123, indicating that the differentiation between IDN and QLD was greater than that between IDN and PNG. Based on the geographical distribution of the three regions (**Figure 3A**), we speculated that QLD was the origin of *E. pellita*, which spread from QLD to PNG and then to IDN.

**Table 2:** The 1^st^ generation of *E. pellita* group as the major source of differentiation index (Fst).

IDN *vs* PNG			IDN *vs* QLD			PNG1 *vs* QLD			PNG2 *vs* QLD		
A17854 vs	A18197^*^	0.028	A17854 vs	A17860	0.120	A18197 vs	A18314	0.070	S16120 vs	A18314	0.197
	A18199^*^	0.021		A17861	0.127		A18596	0.090		A18596	0.226
	A20659^**^	0.041		A18314	0.136		A18597	0.062		A18597	0.195
	S16120^**^	0.067		A18596	0.158		A18598	0.069		A18598	0.199
	S16121^**^	0.042		A18597	0.129		A18599	0.067		A18599	0.176
	S16122^**^	0.033		A18598	0.137		A18749	0.025		A18749	0.094
				A18599	0.123		A18750	0.070		A18750	0.179
				A18749	0.047		A18772	0.073		A18772	0.184
				A18750	0.125		A18773	0.076		A18773	0.197
				A18772	0.131		A18774	0.068		A18774	0.171
				A18773	0.141		A17860	0.063		A17860	0.174
				A18774	0.120		A17861	0.065		A17861	0.179
				B10	0.132		B10	0.069		B10	0.194
				S11947	0.120		S11947	0.066		S11947	0.169
				S14211	0.115		S14211	0.066		S14211	0.164
				S14339	0.106		S14339	0.074		S14339	0.150
	Average	0.039		Average	0.123		Average	0.067		Average	0.178

The provenances of PNG were divided into PNG1 and PNG2, of which 2 provenances with * were PNG1 and 4 provenances with ** were PNG2. One provenance of PNG1 and PNG2 was respectively listed in the table with the Fst of QLD.

According to the Fst results, PNG was divided into PNG1 and PNG2. The Fst between PNG1 (including provenances A18197 and A18199) and QLD was 0.067 and 0.102, respectively, with an average of 0.085, while the Fst between PNG2 (including provenances A20659, S16120, S16121 and S16122) and QLD was 0.175, 0.178, 0.173 and 0.161 respectively, with an average of 0.172, which is twice as much as that between PNG1 and QLD, indicating that the relationship between PNG and QLD wasn’t single. If PNG provenances were only transmitted from QLD, there would be no significant difference between them and the Fst of QLD. Combined with the speculation of Harwood (1998), who observed the characters of *E. pellita* from various sources and speculated that PNG and QLD were two independent populations, we concluded that QLD and PNG2 were two independent origins of *E. pellita*, and that PNG1 was transmitted from QLD. IDN is transmitted from PNG, but it is more likely to be transmitted from PNG1 based on the perspective of Fst coefficient.

The alternative hypothesis holds that PNG2 is the only source, which then spreads to PNG1 and QLD. In this case, the average Fst of PNG2 and QLD, as well as the average Fst of PNG2 and PNG1, are not that different. However, this theory is invalid since PNG and QLD are independent based on the observation of phenotypic traits.

The value range of Fst is 0-1. The maximum value of 1 indicates that the two populations are completely differentiated, while the minimum value of 0 indicates that there is no differentiation between the two populations. It is generally believed that genetic differentiation among populations is very small when Fst is 0-0.05. Between 0.05 and 0.15, there was moderate genetic differentiation among populations. At 0.15 to 0.25, there was a large genetic differentiation among populations. There was large genetic differentiation among populations above 0.25 (Weir and Cockerham, 1984). According to the average Fst values of the first-generation population from all major sources in this study, there was a large differentiation between QLD and PNG2, a moderate differentiation between QLD, PNG1, and IDN, and a small differentiation between other sources.

### 3.5 Linkage disequilibrium analysis

**Figure 6:**
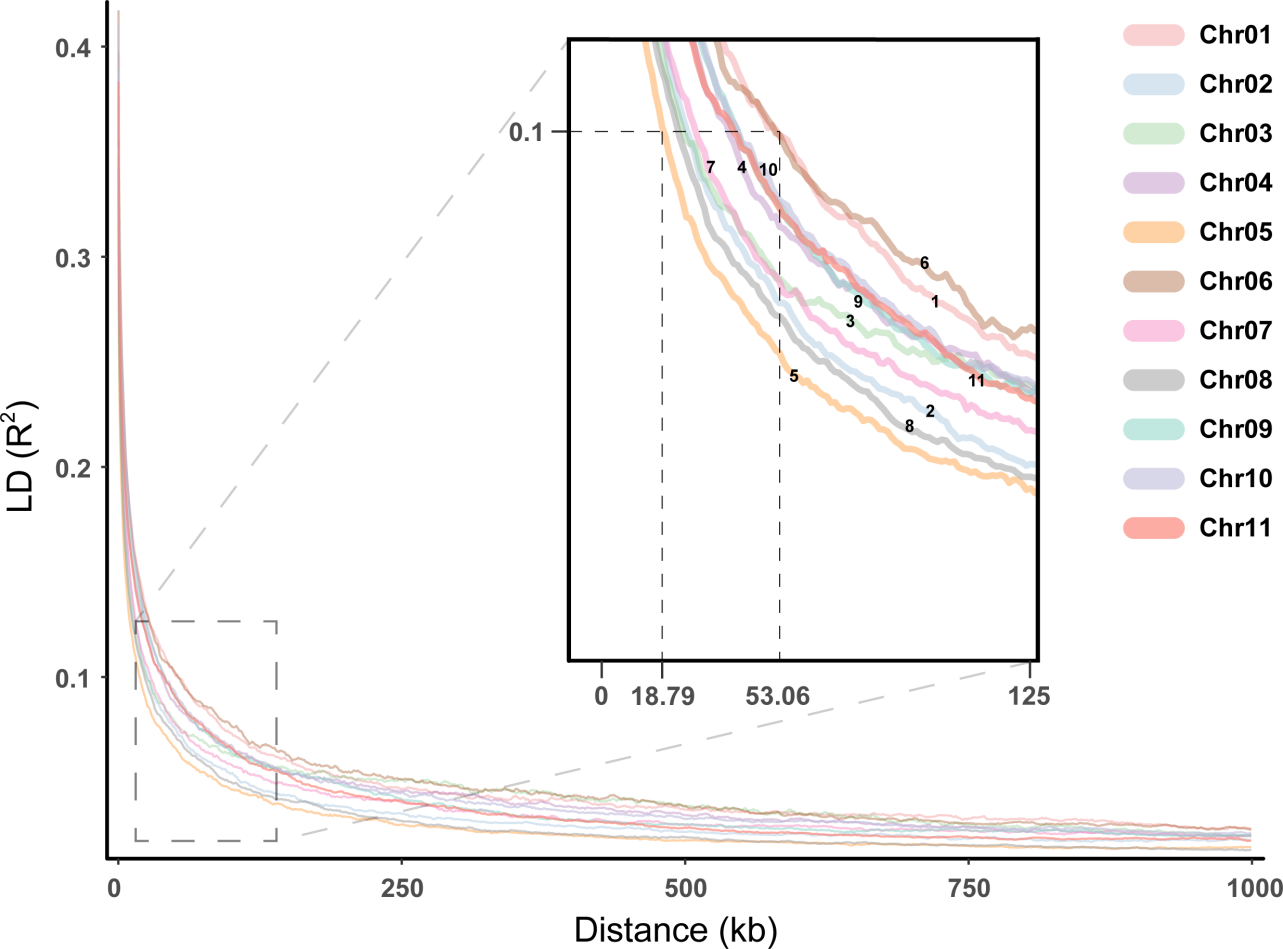
Linkage disequilibrium for the *E. pellita* chromosomes. Different chromosomes were represented in different colors, and the enlarged image was used to clearly show the decay rate of different chromosomes.

Association maps based on linkage disequilibrium (LD) provide a powerful strategy for genetic profiling of complex quantitative traits. The distance to threshold decay (R^2^ ≈ 0.2), and half-maximum decay (R^2^ = 0.1) was calculated within all contigs and all samples in a global analysis, with genome-wide LD decaying to the threshold level within 5.8098 kb, to half-maximum within 33.6428 kb. In addition, the distance of chromosomal average LD decay to the threshold level ranged from 4.5364 to 7.1236 kb, and the distance to the half-maximum ranged from 18.7877 to 53.0599 kb (**Figure 6**). Among these chromosomes, Chr05 showed a faster LD decay, while Chr01 showed a slower LD decay.

### 3.6 Genetic relationship analysis of *E. pellita* population

**Table 3** shows the results of AMOVA for the 196 *E. pellita* using 12,475 SNP markers. The results showed that the genetic variation among population accounted for only 10% of the total variation, while the genetic variation within the populations accounted for 90%.

**Table 3:** Analysis of molecular variance (AMOVA) results.

Variation	Degree of freedom	Sum of squares	Mean of squares	Estimate of variation	Percentage variance
Among Pops	2	8702.456	4351.228	83.044	10%
Within Pops	193	150030.482	777.360	777.360	90%
Total	195	158732.939		860.404	100%

All Fst comparisons between populations showed the values between PNG and QLD were higher than others, while Nei’s genetic distance values between IDN and QLD were higher than others (**Table 4**). In general, Nei’s genetic distance is more suitable for the study of genetic diversity within a population. Thus, a smaller genetic difference (Fst = 0) between IDN and QLD indicates the presence of higher gene flow or similarity in gene frequencies.

**Table 4:** Pairwise PhiPT values (below diagonal) and Nei’s minimum genetic distance (above diagonal) between populations within region for 196 *E. pellita* assessed using GenAlex software.

Population	IDN	PNG	QLD
IDN		0.012	0.024
PNG	0		0.018
QLD	0.057*	0.102**	

* indicated P < 0.05, ** indicated P < 0.01.

A mantel test was performed to obtain a correlation coefficient between genetic distance and geographic distance of *E. pellita*. The SNP marker-based correlation coefficient was Rxy 0.09 (P < 0.05), indicating a positive correlation between genetic distance and geographical location.

## 4 Discussion

### 4.1 SNP annotation

In the coding region, the ratio of non-synonymous to synonymous coding mutations was 0.83, which was similar to that of *Arabidopsis thaliana* (0.83) (Clark et al., 2007), but lower than that of *Cajanus cajan* (1.18) (Varshney et al., 2017) and Chinese plum (1.32) (Wei et al., 2021), indicating that there were fewer mutations causing protein changes in the coding region compared with other species. The ratio of non-synonymous to synonymous coding mutations is less than 1, suggesting that negative selection is an important evolutionary force affecting *E. pellita*.

### 4.2 Analysis of *E. pellita* population structure

The genetic structure of *E. pellita* was analyzed using various methodologies including population structure analysis, PCA, and phylogenetic tree construction, thereby providing complementary information. The 1^st^ generation of *E. pellita* families from three sources: QLD, PNG, and IDN. According to the analysis results, the 23 1^st^ generation provenances can be divided into three large groups. While the division of these groups is not fully compatible with the three large *E. pellita* sources, the families that come from the same source are essentially able to get together. This rule was also supported by the findings of the genetic distance calculation, which showed that there was a significant genetic distance between provenances in different locations and that there was a correlation between genetic distance and geographic position. Similarly, the clustering results of *E. moluccana* and its putative subspecies are not entirely consistent with geographical distribution (Flores-Rentería et al., 2021).

### 4.3 Analysis of genetic diversity

Genetic diversity analysis is of great significance to the evaluation and utilization of plant germplasm resources and the breeding of new varieties. It is also an important component of plant genetics, breeding, conservation, and evolution. In recent years, the majority of studies on the genetic diversity of *Eucalyptus* plants have primarily focused on SSR molecular markers, with a lack of emphasis on SNP markers. However, it is important to note that different molecular marker technologies yield varying results. For instance, when using SSR molecular markers, the He value for *E. Cloeziana* is 0.682 (Lv et al., 2020); whereas when utilizing SNP molecular markers, the He value ranges from 0.2677 to 0.3487 for *E. urophylla* (Yang et al., 2020). Similarly, the genetic diversity value (He) of SSR markers was significantly higher than that of SNP markers in palm trees (Jara-Arancio et al., 2022). These findings highlight the influence of different molecular labeling techniques on parameter values.

It is generally considered that PIC ≥ 0.5 is highly polymorphic, 0.25 ≤ PIC < 0.5 is moderately polymorphic, and PIC < 0.25 is low polymorphic (Botstein et al., 1980). Previous studies based on SSR markers showed that the PIC values of *E. urophylla* (Lu et al., 2018), *E. camaldulensis* and *E. tereticornis* (Arumugasundaram et al., 2011) were highly polymorphic (greater than 0.5). In this study, the mean values of total He and PIC of the 1^st^ generation of *E. pellita* were 0.2502 and 0.2027, indicating a moderately low polymorphism according to Botstein’s theory (Botstein et al., 1980). However, the PIC obtained by SNP markers is at a low level, which may be because each SNP is bi-allelic in nature. Theoretically, the maximum PIC of a single SNP is 0.5 (Eltaher et al., 2018). Similarly, other species studied based on SNP markers had lower PIC, such as corn (0.19) (Adu et al., 2019), cowpea (0.27) (Seo et al., 2020), sorghum (0.24) (Silva et al., 2020). In conclusion, the genetic diversity of *E. pellita* in this study is theoretically not low.

In addition, the difference between the observed heterozygosity and the expected heterozygosity reflected the rationality of SNP markers selection and the genetic diversity of population (Liu et al., 2022). In this study, the He and Ho difference of the three origins (QLD、IND、PNG) of the first-generation population was small, which could accurately estimate the genetic structure of the *E. pellita* population, indicating QLD had a relatively high genetic richness than others.

### 4.4 Analysis of genetic differentiation

Through to the 1^st^ generation group differentiation index (Fst) analysis, this study for *E. pellita* has two natural origins (QLD and PNG) that provided evidence, at the same time, this study also continued to develop the inference, considered one of the big provenances PNG1 spread by big provenance QLD, this assumption is supported by other index, for example, genetic distance. Population differentiation index is one of the important indexes to analyze the origin of species. Many scholars use this index to analyze the origin of species. According to the analysis of *Cajanus cajan* (Varshney et al., 2017), South Asian and sub-Saharan African populations had the lowest Fst values (0.102) compared to South Asian and South American populations (0.126) and South Asian and Central American populations (0.167). Fst values correlated with geographical distance between populations. These Fst values suggest that dal spread from South Asia to sub-Saharan Africa and finally to South and Central America.

The average Fst of the 1^st^ generation natural *E. pellita* population was 0.0913, while the Fst of the 2^nd^ and 3^rd^ generations of *E. urophylla* based on SSR markers was 0.03 (Lu et al., 2018). It can be seen that the Fst of natural populations of *E. pellita* was in the middle level. Similarly, there were *Eucalypts* with low genetic differentiation index, for example, *E. camaldulensis* with Fst values of 0.044-0.065 (Butcher et al., 2002), *E. globulus* was only 0.08 (Jones et al., 2002), *E. marginata* was 0.045 (Wheeler et al., 2003). But some of the other *Eucalyptus* species had higher Fst values, for example, the Fst of *E. curtisii* was 0.30 (Smith et al., 2003), *E. angustissima* was 0.136 (Elliott and Byrne, 2004). *E. morrisbyi* was 0.19 (Jones et al., 2005).

### 4.5 Genetic relationship analysis of three population

The number of SNPs determines the accuracy and reliability of the results. A dataset consisting of 12,745 SNPs was employed for analysis. In comparison to recent studies on other tree species, such as avocado (384 SNPs) (Wienk et al., 2022) and palm (1038 SNPs) (Jara-Arancio et al., 2022), a larger number of filtered SNPs was used in this study, despite their original datasets containing millions or even billions of SNP loci. Thus, differences in analysis results between populations can be attributed to different filtering parameters applied to SNPs.

Considering the filtered SNP loci, AMOVA reveals that 90% of the variation is within populations. This percentage is notably higher compared to other tree species. For instance, in the case of the Fujian tea tree, 66% of the variation is within populations, but these samples have a closer geographic distribution (Liu et al., 2022). In this study, particularly for the QLD population, samples from different provenances span a larger geographic range, leading to greater differences within populations. This highlights a certain correlation between the geographic distribution of provenances and population variation.

To validate the potential association between different populations, Fst and Nei’s distance were employed to assess genetic differences between populations. In this study, Fst values between PNG and QLD were relatively high, while Nei’s distance values between IDN and QLD were higher. This suggests that different analysis methods can yield distinct results. This phenomenon has been observed in other species as well (Wienk et al., 2022; Osundare et al., 2023), possibly attributed to either lower levels of population differentiation or the presence of complex genetic structures. In addition, the Mantel analysis results indicated a moderate correlation between the molecular matrix and the geographic matrix, with a Mantel test value of only 0.09 in this study. For comparison, the *Pinus bungeana* population in central China showed a significantly higher Mantel test value of 0.6 (Tian et al., 2022), and the rice population in northern India exhibited a value of 0.525 (Choudhury et al., 2023). A commonality in these cases is that the samples have relatively concentrated local distributions. As previously reported (Tian et al., 2022), introducing a more dispersed population would lead to a decrease in Mantel test values. Therefore, despite the broad geographic distribution of *E. pellita*, there is still a discernible correlation with genetic distance.

## 5 Conclusion

Although the provenance differences of the 1^st^ generation of *E. pellita* have been indirectly studied by predecessors, the genetic diversity, population structure and genetic differentiation based on SNP markers in *E. pellita* have not been studied. In this study, we conducted rigorous whole-genome resequencing of 196 families of *E. pellita*. A series of analyses were carried out based on the quality screening SNP, and it was concluded that the 1^st^ *E. pellita* population could be divided into three groups. The IDN and PNG provenances were classified into one group, and the QLD provenances were divided into two groups, and the QLD provenances had high genetic diversity. Our analysis of genetic differentiation reveals that QLD was the origin of *E. pellita*. Analyzed 12,475 screened SNPs, showed that the genetic variation among the population accounted for only 10% of the total variation, while the genetic variation within the populations accounted for 90%. The results of this study help to clarify the population structure and provide a valid inference for the population origin of *E. pellita*. The results lay the foundation for genome-wide association analysis.

## Data availability statement

The datasets presented in this study can be found in online repositories at the link below: https://www.ebi.ac.uk/eva/?eva-study=PRJEB70965.

## Author contributions

CW: Conceptualization, Data curation, Writing – original draft. JLa: Investigation, Writing – review & editing. JW: Investigation, Writing – review & editing. WH: Software, Writing – original draft. WL: Investigation, Methodology, Writing – review & editing. YL: Investigation, Project administration, Writing – review & editing. JLu: Conceptualization, Supervision, Validation, Writing – review & editing.
